# High Performance All-solid Supercapacitors Based on the Network of Ultralong Manganese dioxide/Polyaniline Coaxial Nanowires

**DOI:** 10.1038/srep17858

**Published:** 2015-12-08

**Authors:** Junli Zhou, Lin Yu, Wei Liu, Xiaodan Zhang, Wei Mu, Xu Du, Zhe Zhang, Yulin Deng

**Affiliations:** 1 Faculty of Chemical Engineering and Light Industry, Guangdong University of Technology, Guangzhou 510006, Guangdong, China; 2 School of Materials Science & Engineering, Georgia Institute of Technology, Atlanta, GA 30332, USA; 3 School of Chemical & Biomolecular Engineering, Georgia Institute of Technology, Atlanta, GA 30332, USA

## Abstract

In recent years, thin, lightweight and flexible solid supercapacitors are of considerable interest as energy storage devices. Here we demonstrated all-solid supercapacitors (SSCs) with high electrochemical properties, low self-discharge characteristics based on manganese dioxide/polyaniline (MNW/PANI) coaxial nanowire networks. The synergistic effect of MnO_2_/PANI plus the unique coaxial nanostructure of the ultralong nanowires with a highly interconnected network effectively enhance the conductivity and capacitive performance of the SSCs device. The MNW/PANI composite with 62.5% MnO_2_ exhibits an outstanding areal specific capacitance reaching 346 mF/cm^2^ at 5 mV s^−1^ which is significant higher than most previously reported solid supercapacitors (15.3 mF/cm^2^–109 mF/cm^2^) and is close to the that of the best graphene films solid state supercapacitors (372 mF/cm^2^). In contrast, only 190 mF/cm^2^ of areal specific capacitance was obtained for the pure MnO_2_ NW network. The supercapacitors also exhibited low leakage current as small as 20.1 μA, which demonstrated that the MNW/PANI SSCs have great potential for practical applications.

The design and synthesis of nanostructure materials have attracted much attention because of their novel properties and potential applications in catalyst, environment protection, sensors, and energy storage devices. Nowadays, various nanostructure materials have been synthesized by various strategies, such as peptide-directed simultaneous, layer-by-layer (LbL) assembly technique and biomineralization etc^1^,^2^. However, it still remains a great challenge to develop facile and economic synthetic methods for the construction of hierarchical architectures with novel tunable material properties.

Energy storage devices, such as Li-ion batteries, fuel cells and supercapacitors, are considered as an important part of the clean energy. Among the existing energy storage devices, much effort has been devoted to lightweight and flexible supercapacitors due to their fast charge and discharge ability, high power density and long life cycles^3^,^4^,^5^,^6^. Up to now, many supercapacitor materials focused on different carbon-based nanomaterials, such as graphene^7^,^8^, carbon cloth^9^, carbon nanotube (CNTs)^10^, etc. Moreover, surface modification of carbon based materials using pseudocapacitor materials such as conducting polymer and metal oxides can effectively enhance their electrochemical performance^11^,^12^. Especially, the graphitic carbons after chemical modification/doping have novel tunable properties, including the controllability of electronic energy level, charge density, surface energy and surface reactivity, which benefit for flexible electronics/optoelectronics, energy conversion/storage. However, the inert chemical structures with low surface energy for CNTs and graphene are the principal bottleneck for cost-effective solution processing^13^.

Pseudocapacitive-type electrode materials such as transition metal oxides and conductive polymers have attracted much attention due to their low cost but high electrochemical performance. Transition metal oxides and conducting polymers such as manganese dioxides (MnO_2_) and polyaniline (PANI) have been found to exhibit excellent pseudocapacitive behavior^14^,^15^,^16^,^17^. Among the transition metal oxides, manganese dioxide (MnO_2_) with different morphologies has been considered as an alternative electrode material due to the low cost, high theoretical specific capacitance (1370 F g^-1^) and environmental friendly energy conversion^18^,^19^. Unfortunately, the poor electronic conductivity of MnO_2_ (10^−5^ to 10^−6^ S cm^−1^)^20^ may cause the decrease in the specific capacitance and power density of supercapacitors during high-rate charge-discharge process. In order to improve the electrochemical performance of MnO_2_, much effort has been made to fabricate composite materials with good electrical conductivity materials even high cost noble metals^21^,^22^. Conducting polymers, such as PANI has been extensively studied as electrode materials because of their ease of synthesis, good electrical conductivity, high chemical stability, high doping properties and low cost. Heterostructured nanomaterials have been attracted particular attention because the synergic properties and functionalities of these combined materials enable this composite material to exhibit a high electrochemical performance. Sumboja *et al.* have prepared manganese dioxide/polyaniline coaxial nanowires by self-terminated double surfactant polymerization for pseudocapacitor electrode, showing specific capacitance of 498 F g^−1^ in Na_2_SO_4_ electrolyte^23^. Jaidev *et al.* reported a novel binary hybrid nanocomposite based on polyaniline (PANI) and α-MnO_2_ nanotubes (MNTs) and found that the nanocomposite exhibited good electrochemical performance with a specific capacitance of 626 F g^−1^ in H_2_SO_4_ electrolyte^24^. Although high performance MnO_2_ based supercapacitors have attracted much attention due to their higher power density, longer life cycles, faster charge and discharge rate than batteries^25^,^26^,^27^, almost all PANI-MnO_2_ based supercapacitors used electrolyte solution as the charge transfer medium^28^,^29^,^30^. In particular, solid and lightweight supercapacitors have been a new focus of researches^31^,^32^,^33^. Considering the safety issues, the SSCs are superior to the traditional liquid electrolyte supercapacitors, since the liquid electrolyte leakage is a severe issue, especially when the materials are hazardous for human and environment. In this context, a simple and low-cost method to fabricate light weight solid supercapacitors is greatly desired for energy storage devices.

Here we demonstrated the solid state supercapacitors (SSCs) with high electrochemical properties, low self-discharge characteristics and high stability based on manganese dioxide/polyaniline (MNW/PANI) coaxial nanowire networks. Practically, instead of the conventional hydrothermal method using Mn^II^ (MnSO_4_) and oxidizing agent (KMnO_4_, NaClO_3_)^34^,^35^, in this work, MnO_2_ nanosheet-assisted hydrothermal and *in situ* polymerization method was designed to construct the well dispersed MNW/PANI coaxial configuration. Such nanostructured MNW/PANI coaxial configuration with highly interconnected network provides an excellent structure for supercapacitor electrodes, which is favorable for fabricating a high efficient supercapacitor. Besides, a novel and simple method using the polyvinyl alcohol (PVA)/H_3_PO_4_ electrolyte to fabricate the MNW/PANI based SSCs is presented. The fabricated SSCs exhibited high electrochemical performance, which is significantly higher than most flexible supercapacitors reported in literature. And a practical use of the SSCs devices to light up the LEDs demonstrated the great potential in energy storage devices.

## Results and Discussion

**Figure 1:**
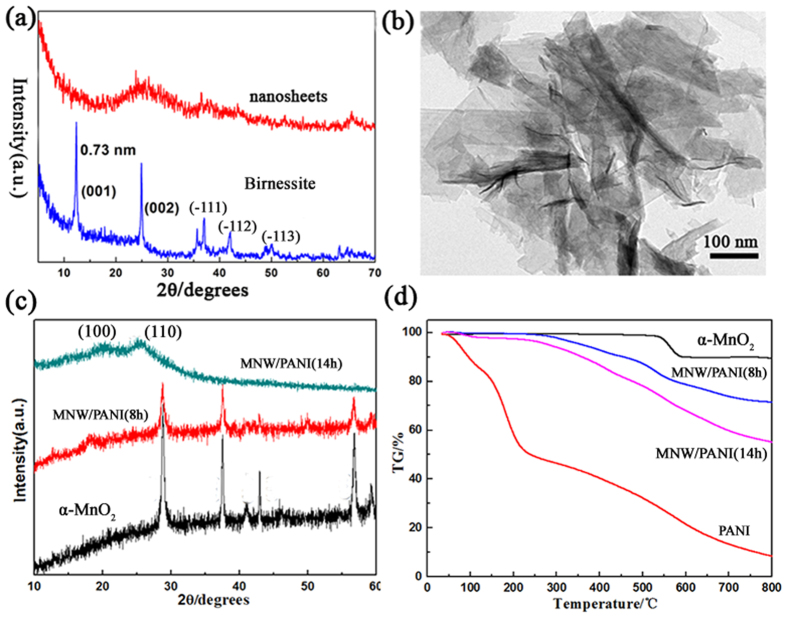
(**a**) XRD patterns for Birnessite type MnO_2_ and nanosheets. (**b**)TEM image for MnO_2_ nanosheets. (**c**) XRD patterns and (**d**) TGA curves for α-MnO_2_ nanowires, PANI, MNW/PANI(8h), MNW/PANI(14 h).

**Figure 2:**
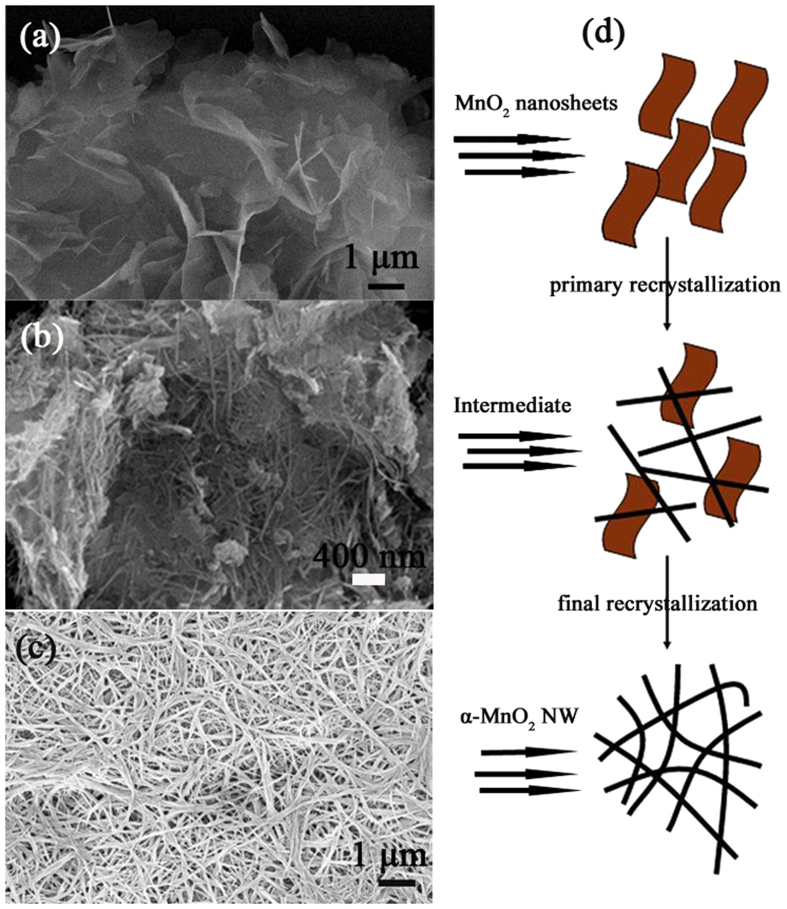
Schematic illustration of the formation from 2D Nanosheet to 1D Nanowires.

The ultralong MnO_2_ NW were prepared first via one-pot reactions of MnO_2_ nanosheets and KClO_3_ at 160 °C for 12 h (Supporting Information Experimental Section). MnO_2_ nanosheets were prepared using the same method as previously reported^36^. As shown in Fig. 1a, the Birnesstie type δ-MnO_2_ showed two diffraction peaks which could be indexed as (001) and (002), respectively. After the ultrasonic treatment with TMAOH, the absence of reflections peaks indicated the loss of the original crystalline structure, suggesting that the Birnesstie type δ-MnO_2_ was delaminated to the individual nanosheets^37^. TEM image showed that the nanosheets are extremely thin, which looks like graphene (Fig. 1b). It was interesting to find that these thing δ-MnO_2_ nanosheets could be further converted to α-MnO_2_ ultralong nanowires. The XRD results of α-MnO_2_ NW are shown in Fig. 1c, exhibiting pure tetragonal phase (space group *I4/m*) of α-MnO_2_ with lattice constants of a = 9.7847 Å, b = 9.7847 Å and c = 2.8630 Å (JCPDS No.44-0141). The morphological evolution of Two-Dimensional (2D) MnO_2_ nanosheets and their shape transformation to One-Dimensional (1D) ultralong MnO_2_ NW was investigated by time-dependent SEM images, as shown in Fig. 2. At the initial stage, δ-MnO_2_ nanosheets were observed and they were gradually changed into 1D nanowire morphology with the progress of reaction time. Li *et al.* have reported the rolling growth mechanism for δ-MnO_2_ nanosheets to form the α-MnO_2_ NW^38^. However, in our study, no curling or rolling of the nanosheets was observed (Fig. 2b). Although the actual mechanism of conversion of δ-MnO_2_ nanosheets to α-MnO_2_ NW in our experiment is not clear, it is believed that the process obeys a recrystallization process which may be similar to the previous report^39^. In the recrystallization process, the new α-MnO_2_ nuclei were first formed in the solution. The δ-MnO_2_ nanosheets dissolute and recrystallized as 1D nanowires. The diameter and length of the nanowires increase with time and finally form the ultralong nanowire networks (Fig. 2c).

The PANI/MnO_2_ coaxial nanowires were then obtained by using the α-MnO_2_ NW as the oxidant template to initiate the polymerization of aniline in H_2_SO_4_ solution (Supporting Information Experimental Section). A series of time-dependent experiments was conducted. The XRD patterns for MNW/PANI still exhibited α-MnO_2_ phase with reduced intensity of MnO_2_ characteristic peaks for 8 h. The reduced diffraction intensity is because that the PANI layer can cause the X-ray scattering. After the extension of the reaction time to 14 hours, α-MnO_2_ as oxidant and template was almost removed. No α-MnO_2_ peaks were observed (Fig. 1c). Two peaks at 2theta 20° and 25° shown in Fig. 1c represent the periodicities parallel (100) and perpendicular (110) to the PANI chain respectively^40^. PANI coating on MnO_2_ NW was also successfully confirmed by thermogravimetric (TG) measurements (Fig. 1d). For comparison, we have also prepared MnO_2_ NW and PANI powder. From the TG curves, it can be inferred that the mass of MnO_2_ in the composite MNW/PANI (8h) and MNW/PANI (14h) is about 81% and 62.5%, respectively.

**Figure 3:**
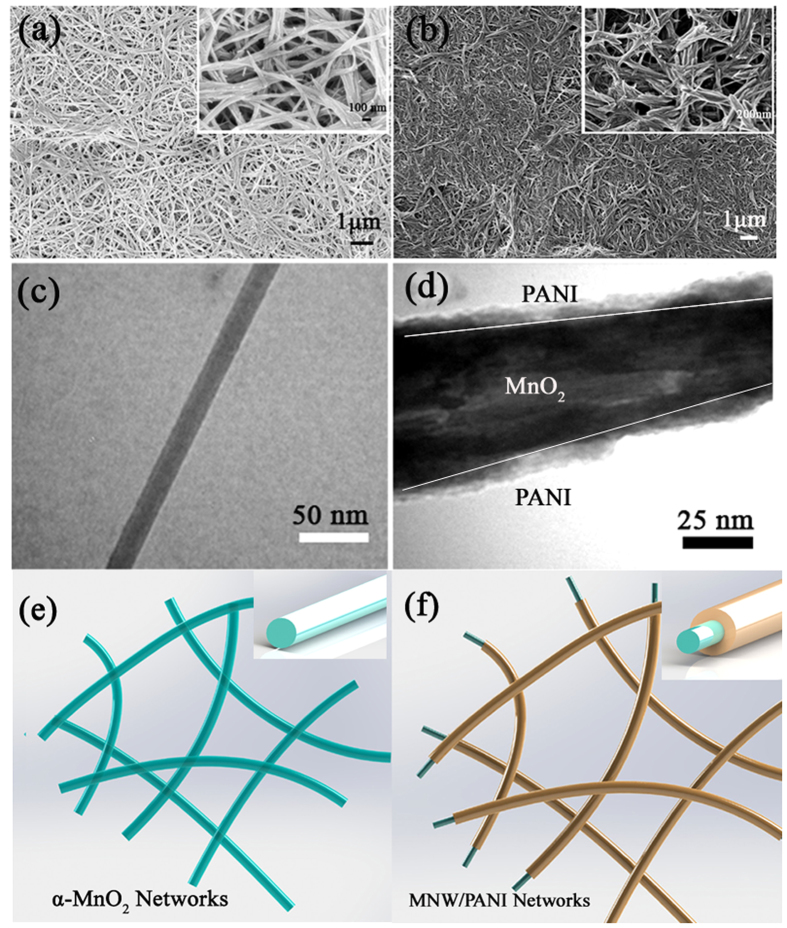
SEM images of (**a**) α-MnO_2_, (**b**) MNW/PANI (8 h). TEM images of (**c**) α-MnO_2_ NW, (**d**) MNW/PANI (8 h). (**e**) Schematics of *In situ* polymerization of aniline based α-MnO_2_ NW.

The ultralong MnO_2_ nanowires are shown in Fig. 3a. The actual length of the MnO_2_ NW is too long to be measured from SEM image. The SEM and TEM images (Fig. 3a,c) indicate that the diameter of the MnO_2_ NW is about 50 nm. The α-MnO_2_ NW free-standing membrane with a good mechanical stability could be prepared by simply filtrating the NW suspension on a filter paper (Figure S1). The nanowires are physically entangled and interconnected in the porous network membrane. Because α-MnO_2_ NW is an oxidization agent that can initiate the polymerization of aniline, when it was immersed into aniline solution, polymerization started on the surface of MnO_2_ NWs. If the concentration of aniline monomer in the solution is low, a uniform and thin PANI coating layer on α-MnO_2_ NW was obtained. As the reactions proceeded, PANI shells grew and α-MnO_2_ NW was partially reduced spontaneously. Then the well-dispersed MNW/PANI with uniform interconnection networks was obtained. The networks possess a continuously cross-linked structure with pore sizes in the range of sub-micrometer to several micrometers (Fig. 3b). Such network structure will be beneficial for filtrating solid electrolyte that can transport electrons between two MnO_2_/PANI electrodes in a supercapacitor. Uniform coating of PANI has been observed on MnO_2_ NW, demonstrating the formation of MNW/PANI coaxial nanowires (Fig. 3d). Successful PANI coating on MnO_2_ NW surface is also confirmed by FTIR (Figure S2 in supporting information). The bands around 523 and 465 cm^−1^ in both spectrums can be assigned to the Mn–O stretching vibration. Bands at 1569 and 1482 cm^−1^ in MNW/PANI coaxial NW is due to the stretching vibration of quinoid and benzenoid ring, indicating that the resultant PANI was in its emeraldine state^23^. The peak around 1292 cm^−1^ is corresponding to C–NH^ + ^stretching (characteristic of the polaron form of PANI emeraldine salt) and the strong peak around 1132 cm^−1^ is the characteristic peak of PANI conductivity^24^. FTIR spectrum of the MNW/PANI coaxial NW shows characteristic bands of PANI as well as of MnO_2_, which confirms the presence of both components in the coaxial heterostructures.

The designed MNW/PANI interconnection porous networks combined the advantages of high theoretical specific capacitance of α-MnO_2_ NW and the good electrical conductivity of PANI. To evaluate the electrochemical activity of MNW/PANI materials, solid supercapacitor devices were assembled using MNW/PANI as the working electrodes and PVA/H_3_PO_4_ as solid electrolyte. The working electrodes were fabricated by compressing a mixture of the MNW/PANI-acetylene black- polytetrafluoroethylene (PTFE) with a weight ratio of 0.85:0.15:0 on Ni foam at 0.2 MPa. The dimension of the electrode is 1 cm × 1 cm. Two symmetric pieces of identical MNW/PANI electrodes and a separator (filter paper, Whatman Corporation) were immersed into the electrolyte (PVA/H_3_PO_4_) for 10 min, and then taken out to fabricate as a sandwich type of supercapacitor (Figure S3). Ni foam was used as both a current collector and a mechanical support due to the high conductivity and the instinct macroporous structure to hold the electrode materials^41^,^42^,^43^.

**Figure 4:**
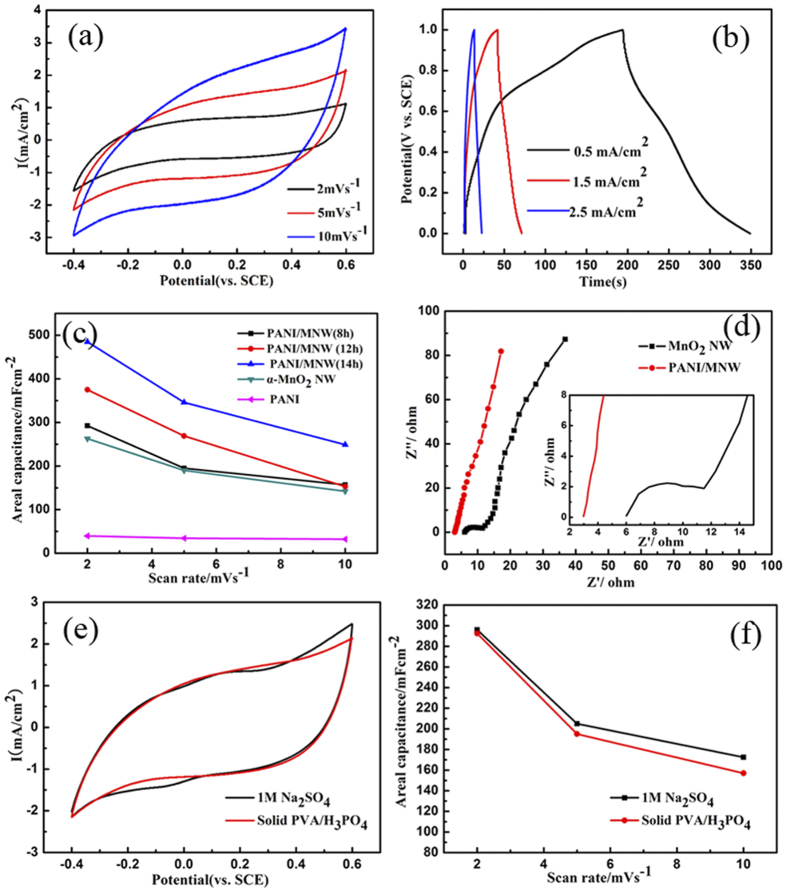
(**a**) Cyclic voltammograms (CV) and (**b**) galvanostatic charge/discharge curves of solid supercapacitor for MNW/PANI (8h). (**c**) Specific areal capacitance C_s_ vs scan rates for different PANI deposition time, ranging from 8 to 14 h. (**d**) Nyquist plots of MNW/PANI and MnO_2_ NW. (**e**) CV at a scan rate of 5 mV s^−1^ and (**f**) Specific areal capacitance C_s_ vs scan rates curves of MNW/PANI (8 h) in 1 M Na_2_SO_4_ solution and in PVA/H_3_PO_4_ solid electrolyte.

Cyclic voltammetry (CV) measurements were performed to test the electrochemical properties of the designed MNW/PANI networks as electrode materials using a three-electrode cell. Figure 4a shows that the CVs for MNW/PANI (8h) over a range of scan rates of 2, 5, 10 mV/s, with a potential window of −0.4−0.6 V versus a saturated calomel electrode (SCE). The CV curves at low scan rate show a nearly rectangular shape, indicating good electrochemical capacitive behavior of the SSCs. Although the CV curves were somewhat distorted when the scan rate increases up to 10 mV/s, the SSCs still exhibited good electrochemical capacitance performance. The areal capacitances can be calculated from the CV curves according to C_s_ = I/ (2 · s · ν), where s is the area of one electrode sheet, ν is the potential sweep rate, and I is the applied current defined by I = A/ΔV, where A present the integrating area of the curves and ΔV is the voltage difference. The areal capacitances calculated from the CV profiles decreased from 293 to 157 mF/cm^2^ with the scan rate rise from 2 to 10 mV/s. The decrease of capacitance is mainly because that the redox reactions of insertion-deinsertion of electrolyte are a time-dependent process^44^,^45^.

Galvanostatic charging/discharging tests were conducted under a stable potential window of 0–1.0 V at different current densities of 0.5, 1.5 and 2.5 mA/cm^2^, and the results are shown in Fig. 4b. It was observed that the discharge curves at 0.5 mA/cm^2^ show two plateaus at about 0.7 V and 0.3 V, respectively. The higher one is due to oxidation of Emeraldine Salt (ES) to pernigraniline, and the lower one is further oxidation of Mn(III) to Mn(IV). Moreover, the discharge profiles of the electrodes are not standard straight lines, but somewhat curved in nature, exhibiting a pseudocapacitive characteristic. The areal capacitance of MNW/PANI supercapacitors with different PANI deposition time and different scan rates are given in Fig. 4c. For comparison, the C_s_ for the bare ultralong α-MnO_2_ NW was also tested and calculated to be 263 mF/cm^2^ at 2 mV/s. The good capacitive behavior of MnO_2_ is due to the fast and reversible surface redox reactions of MnO_2_^46^. The C_s_ improves significantly after coating a layer of PANI. The calculated C_s_ of MNW/PANI (14h) at 2 mV/s (485 mF/cm^2^) is much higher than pure α-MnO_2_ NW (263 mF/cm^2^) and pure PANI (39.75 mF/cm^2^) which indicates the coordinated contribution of the pseudocapacitance effect of PANI coated on the MnO_2_ NW. Moreover, the C_s_ for MNW/PANI increases with the increase of deposition time due to the increasing amounts of PANI. Meanwhile the C_s_ also increases with the decrease of scan rates due to more adequate Faradaic reactions at lower scan rates^47^. The C_s_ for MNW/PANI (14 h) supercapacitors was calculated to be 346 mF/cm^2^ at the scan rate of 5 mV/s, which is significant higher than most previously reported solid supercapacitors (15.3 mF/cm^2^–109 mF/cm^2^)^32^,^48^,^49^,^50^,^51^ and is close to that of the best graphene films solid state supercapacitors (372 mF/cm^2^)^7^. The enhanced capacitance for MNW/PANI in this work can be attributed to several reasons. Firstly, the well dispersed nature and the highly mesoporous structure of the designed MNW/PANI interconnection networks, which facilitates more ions transfer to the porous structure having more redox reactions. As shown in Figure S4 and Table1 (Supporting information), the specific surface area of MNW/PANI (105 m^2^ g^−1^) is lower than MnO_2_ NW (163 m^2^ g^−1^). However, it is interesting that the MNW/PANI has larger pore volume 0.71 cm^3^g^−1^ with large average pore diameter (23.45 nm). Enhancement of internal pore volume may therefore be responsible for increasing the capacitance, which enhances more accessibility to the electrolyte for internal surface adsorption in the electrode. Secondly, after coating a thin layer of PANI, the electric resistance within MnO_2_ NW networks is reduced. The Electrochemical Impedance Spectroscopy (EIS) measurement results are shown in Fig. 4d and the equivalent circuit is present in Figure S5. The equivalent circuit includes the following parameters: equivalent series resistance (Rs), charge-transfer resistance (Rct), double layer capacitance (CDL), pseudocapacitance (Cps) and Warburg behaviour (W). At high frequency range, the value of the intercept at the real axis is used to estimate the equivalent series resistance (Rs) including electrolyte resistance, electrode resistance and the contact resistance between the electrode and electrolyte. The Rs value reduced from 6 to 2.9 Ω compared MNW/PANI with MnO_2_ NW which indicates the uniform PANI coating is electrostatically bonded to the MnO_2_ NW^23^. The formation of mesoporous structure for MNW/PANI in the designed synthesis method is responsible for lower Rs value. The low Rs of the samples help to guarantee the high capacitance. Moreover, no distinct semicircle was observed for the plot of MNW/PANI compared to MnO_2_ NW, which indicates that the MNW/PANI coaxial configuration resulted in the lower charge transfer resistance (Rct) between the active material interface and electrolyte. In addition, Nyquist plot of MNW/PANI SSCs shows a straight line in the low frequency region which is attributable to Warburg impedance (W). The angle between the linear region of the plots and the real axis (Warburg region) resulted from the frequency dependence of ion diffusion and transport in the electrolyte. The angle goes larger after coating PANI which indicates the short ion diffusion path in the electrolyte to the electrode interface (Figure S6). Finally, good coordination between PANI shell and MnO_2_ NW core help to improve the capacitance of MNW/PANI SSCs. PANI shell could be well electrostatically bonded in the MnO_2_ NW interface through *in situ* polymerization using ultralong MnO_2_ NW as oxidant template. As proved in CV curves previously, the C_s_ for MNW/PANI is 1.8 times of pure MnO_2_ NW and 12 times of pure PANI which is not the simple additive effect of PANI and MnO_2_. The comparisons of the as fabricated MNW/PANI supercapacitors in 1 M H_2_SO_4_ solution and in PVA/H_3_PO_4_ solid electrolyte are shown in CV curves at a scan rate of 5 mV/s (Fig. 4e). The two curves show the same patterns with slightly difference. The areal capacitance of 195 mF/cm^2^ at a scan rate of 5 mV/s is very close to the supercapacitor measured at 1 M H_2_SO_4_ electrolyte (205 mF/cm^2^). The nearly overlapped CV curves indicate that the electrochemical properties of MNW/PANI supercapacitors in PVA/H_3_PO_4_ solid electrolyte are almost as good as they are in H_2_SO_4_ aqueous solution (Fig. 4f), which suggests the fast ion transportation of PVA/H_3_PO_4_ solid electrolyte^7^,^33^.

**Figure 5:**
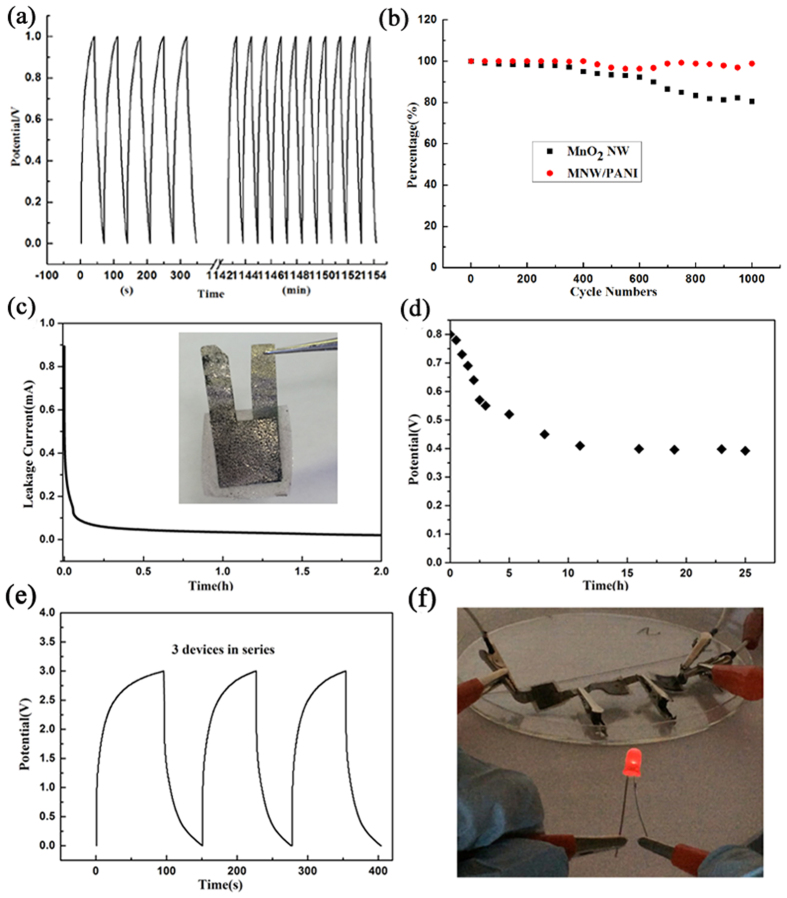
(**a**) Charge/discharge curves of the MNW/PANI device at the current density of 1.5 mA/cm^2^ and (**b**) Cyclic stability for MNW/PANI and MnO_2_ NW device. (**c**) Leakage current curves of the solid device charged at 2 mA to 0.8 V and kept at 0.8 V for 2 h (inset: photographs of the symmetric supercapacitor based on MNW/PANI). (**d**) Self discharge curve of the device after charging at 0.8 V for 15 min. (**e**) Galvanostatic charge/discharge curves at 1.5 mA of the three SSCs connected in series. (**f**) A LED lighted by a device composed of three SSCs connected in series.

The electrochemical stability of the SSCs device was examined under continuous charge/discharge test at the current density of 1.5 mA/cm^2^ for 1,000 cycles (Fig. 5a). The MNW/PANI SSCs device almost kept the same performance in the first 400 cycles test and a slightly fluctuation with 1.5% degradation of the specific capacitance all the way to 1,000 cycles, indicating good stability of the MNW/PANI sample (Fig. 5b). However, the specific capacitance for pure MnO_2_ NW faded significantly with 18% degradation at the end of cycles, which is attributed to the dissolution of MnO_2_ into the electrolyte. Coating a thin layer of PANI on MnO_2_ was observed to improve the long term cycling stability of MnO_2_. For practical application, it is very important to evaluate the self-discharge characteristics and leakage current of the solid supercapacitors which are few discussed in recent reports^7^,^50^. For the leakage current test, the solid device (inset picture in Fig. 5c) was first charged to 0.8 V at 2 mA and kept at 0.8 V for 2 h to acquire the current data. As shown in Fig. 4c, the leakage current decreased significantly in the first 10 min (from 0.9 mA to 57.8 μA) and quickly stabilized at 20.1 μA, which is essentially the leakage current through the device. This value is comparable to that of polyaniline/carbon nanotube composite supercapacitor (17.2μA)^12^, indicating the very small leakage current of the designed SSCs device. The stability of the solid supercapacitors is demonstrated by a self-discharge test represented by the open-circuit voltage versus time course. As shown in Fig. 5d, the solid supercapacitors after being charged at 0.8 V for 15 min underwent a rapid self-discharge process within several minutes and reached a stable output voltage at 0.42 V after 25 h. The result is comparable to previous results of solid supercapacitors (0.3–0.4 V)^7^,^52^. These results demonstrate that the solid supercapacitors in our studies exhibit excellent low self-discharge characteristics, which is a big concern for practical applications in electronics. As shown in Fig. 5f, three SSCs were connected in series to light a light-emitting diode (LED, the lowest working potential is 1.6 V). Every SSCs used here has the same area (1 cm^2^) and mass loading (~8.5 mg for each electrode). The whole series device was evaluated by galvanostatic charge/discharge measurements (Fig. 5e). The potential window extended from 1.0 V for single SSCs to 3.0 V for the series device. After charged at 3 V for 15 s, the series device could light the LED for about 90 s (Fig. 5f and see supporting information, video 1). Electrochemical energy storage is associated with electron transfer. The EIS measurement results indicated that the lower ESR and charge transfer resistance could improve a series of fast, reversible electron-transfer reactions (Figure S7).

In summary, MnO_2_ nanosheet-assisted hydrothermal and *in situ* polymerization method was designed to construct the well dispersed MNW/PANI coaxial configuration. We have successfully fabricated MNW/PANI SSCs with high electrochemical performance. The well dispersed and highly mesoporous of MNW/PANI interconnection networks provided good structure for supercapacitors electrode, and the good interaction between PANI and MnO_2_ NW provided fast electron and charge transportation paths to achieve high capacitance. Higher C_s_ was achieved for MNW/PANI(14 h) reaching 346 mF/cm^2^ at 5 mV s^−1^ as compared to the pristine MnO_2_ NW(190 mF/cm^2^). The self-discharge characteristics and practical use as power source for LED lighting indicated the great potential application in electronics.

## Additional Information

**How to cite this article**: Zhou, J. *et al.* High Performance All-solid Supercapacitors Based on the Network of Ultralong Manganese dioxide/Polyaniline Coaxial Nanowires. *Sci. Rep.* **5**, 17858; doi: 10.1038/srep17858 (2015).

## Supplementary Material

Supplementary Information

Supplementary Video 1
