# Supplementary material for: High Performance All-solid Supercapacitors Based on the Network of Ultralong Manganese dioxide/Polyaniline Coaxial Nanowires
Source: Sci Rep. 2015 Dec 8;5:17858. doi: 10.1038/srep17858 (PMC4672316; doi:10.1038/srep17858)
Supplement: Supplementary Information [file srep17858-s1.doc]

**High Performance All-solid Supercapacitors**

**Based on the Network of Ultralong Manganese dioxide/Polyaniline Coaxial Nanowires**

Junli Zhou^a,c^, Lin Yu*^,a^,Wei Liu^c^, Xiaodan Zhang^b^, Wei Mu^c^, Xu Du^c^, Zhe Zhang^c^, Yulin Deng*^,c^

^a^Faculty of Chemical Engineering and Light Industry, Guangdong University of Technology, Guangzhou 510006, Guangdong, China and ^b^ School of Materials Science &Engineering, Georgia Institute of Technology, Atlanta, GA 30332, USA and ^c^ School of Chemical & Biomolecular Engineering, Georgia Institute of Technology, Atlanta, GA 30332, USA.

*corresponding author：

E-mail: [yulin.deng@chbe.gatech.edu](mailto:yulin.deng@chbe.gatech.edu) (Yulin Deng)

[gych@gdut.edu.cn](mailto:gych@gdut.edu.cn) (Lin Yu)

**Experimental Section**

**Preparation of α-MnO_2_** **Nanowires.** MnO_2_ nanosheets were prepared using the same method as previously reported. The MnO_2_ nanosheets thus obtained were mixed with 0.245 g KClO_3_, 1 mL H_2_SO_4_ and 15 mL distilled water under stirring. The resulting solution was heated at 160 °C for 12 h. The solid precipitate was centrifuged, washed and then dried at 60 °C for 12 h.

**Preparation of PANI/MNW Hybrids.** Aniline solution was prepared by dissolving 0.285 g aniline sulfate in 100mL 1mol/L cool sulfuric acid aqueous solution with magnetic stirring. The monomer solution was then cooled to 0-5 °C in refrigerator. 0.09 g of the as-obtained α-MnO_2_ nanowires was immersed into the cooled monomer solution and kept in refrigerator. The polymerization of aniline was initiated by α-MnO_2_ nanowires at 0-5 °C for 8, 12 and 14 h, respectively. After the reaction, the samples were taken out and washed with deionized water and ethanol, and dried at 60 °C for 12 h.

**Preparation of PANI/MNW Solid-State Supercapacitors.** The PVA/H_3_PO_4_ gel electrolyte was prepared as previous report. The working electrode was fabricated by compressing a mixture of the MnO_2_-acetylene black- polytetrafluoroethylene (PTFE) with a weight ratio of 0.85:0.15:0 on a 1cm×1cm foamed nickel at 0.2 MPa. Two sheets of the working electrodes were immersed into the PVA/H_3_PO_4_ solution for 5 min, keeping the bare Ni foam part above solution, then taken out and assembled together with the separator (glass filter paper, Whatman Corporation) as a sandwich type as shown in Figure 4a. The device was allowed to dry in room temperature overnight before tests.

**Sample Characterizations.** The X-ray diffraction (XRD) patterns of the samples were analyzed by X’Pert PRO diffractometer equipped with a Cu Kα radiation source using an operation voltage and current of 40 kV and 40 mA. Scanning electron microscopy (SEM) was performed in a Digital Scanning Microscope LEO 1530 operated at 3 kV. Transmission electron microscopic (TEM) images were obtained on a JEOL JEM-100CX II transmission electron microscope with an acceleration voltage of 100 kV. Thermogravimetric analysis (TGA) was conducted on a PerkinElmer STA 6000 Thermal Analyzer at 10 °C /min heating rate under nitrogen. Electrochemical performance tests were performed by using an electrochemical station (CHI 660E).

**References**

[1] Y. Cui, Z.-H. Liu, M. Wang, K. Ooi, Chem Lett 35 (2006) 740-741.

[2] L. Yuan, X.-H. Lu, X. Xiao, T. Zhai, J. Dai, F. Zhang, B. Hu, X. Wang, L. Gong, J. Chen, C. Hu, Y. Tong, J. Zhou, Z.L. Wang, ACS Nano 6 (2011) 656-661.


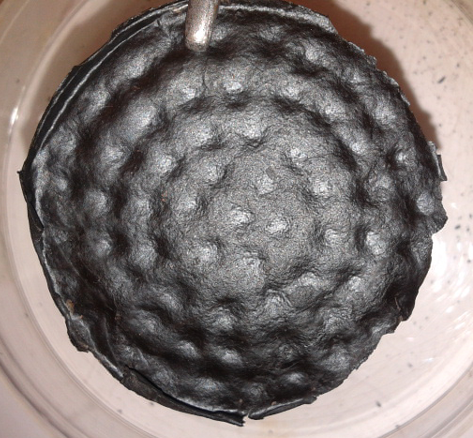


Figure S1. Digital photograph of α-MnO_2_ NW membrane


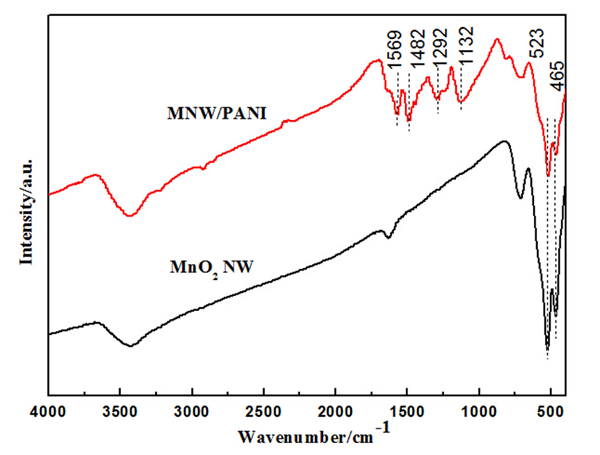


Figure S2. FTIR spectrums for α-MnO_2_ NW and PANI/MNW coaxial nanowires.


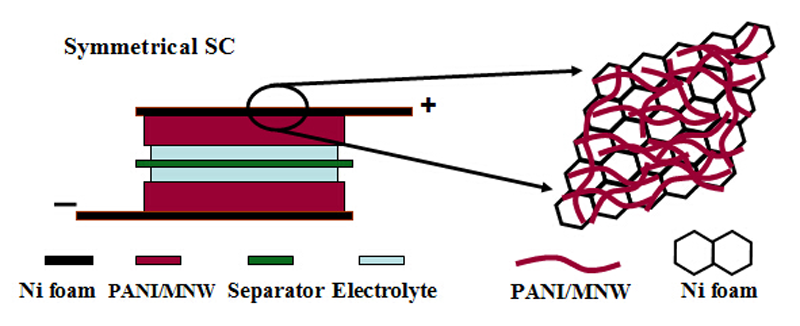


Figure S3. Schematic illustration of the designed symmetric supercapacitor device


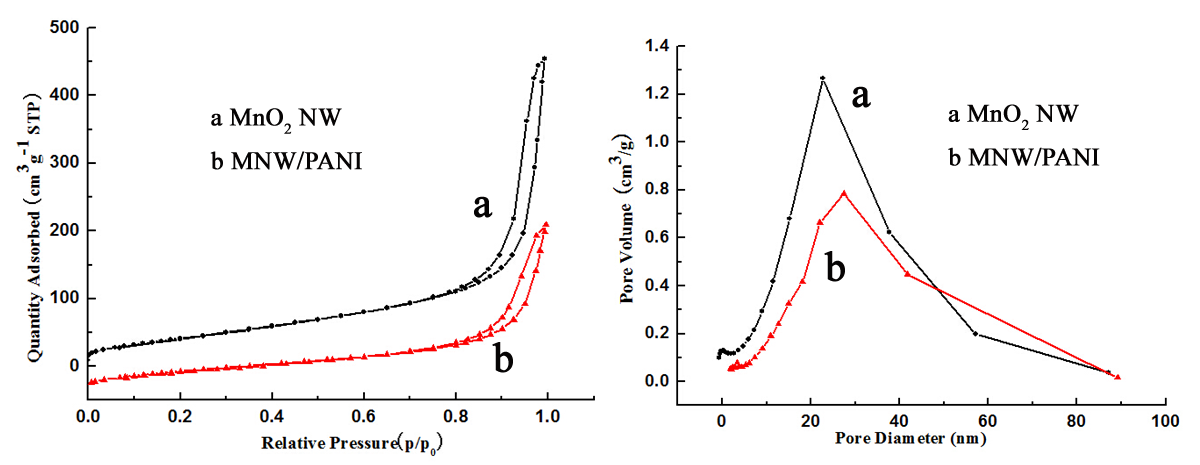


Figure S4. Nitrogen adsorption–desorption isotherms and corresponding pore sizedistribution plots for α-MnO_2_ NW and PANI/MNW coaxial nanowires.

Table1 Specific surface area, pore volume (Vp) and average pore diameter for for α-MnO_2_ NW and PANI/MNW coaxial nanowires.

| Sample S_BET_  V_d_^p^ Average pore (m^2^ g^-1^) (cm^3^g^−1^) diameter (nm) |
| --- |
| MnO_2_ NW 163 0.59 17.19  MNW/PANI 105 0.71 23.45 |


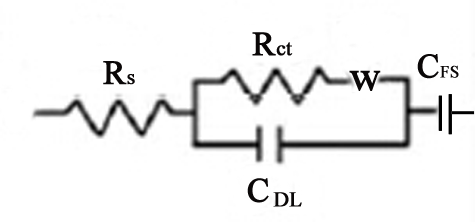


Figure S5. The equivalent electric circuit diagram.


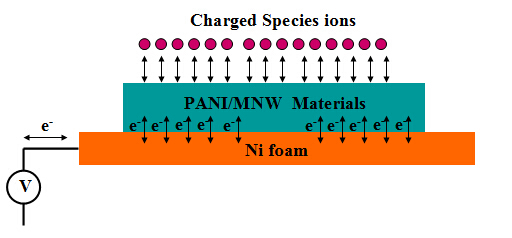


Figure S6. Surface charge transfer mechanism of electrode materials PANI/MNW

**Supplementary Vedio1:** After charged at 3 V for 15 s, the series device could light the LED for about 90 s.


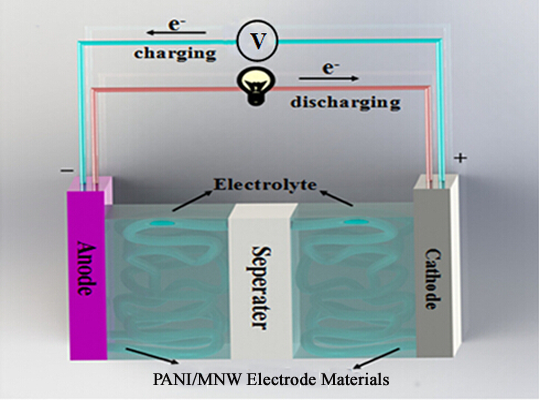


Figure S7. The charge and discharge mechanism for the designed SSCs device.
